# Machine Learning for Valproic Acid Therapy: A Scoping Review of Pharmacokinetic-Prediction Models and Pharmacokinetic-Informed Clinical Outcome Models

**DOI:** 10.3390/pharmaceutics18081005

**Published:** 2026-08-14

**Authors:** Janthima Methaneethorn, Supavadee Aramvith, Wanaporn Charoenchokthavee, Sohaib Habiballah, Khanita Duangchaemkarn, Brad Reisfeld

**Affiliations:** 1 Department of Pharmacy Practice, Faculty of Pharmaceutical Sciences, Chulalongkorn University, Bangkok 10330, Thailand; 2 Multimedia Data Analytics and Processing Research Unit, Department of Electrical Engineering, Faculty of Engineering, Chulalongkorn University, Bangkok 10330, Thailand; supavadee.a@chula.ac.th; 3 Department of Pharmacy, The Faculty of Medicine Vajira Hospital, Navamindradhiraj University, Bangkok 10300, Thailand; wanaporn@nmu.ac.th; 4 Department of Chemical & Biological Engineering, King Abdulaziz University, Jeddah 22254, Saudi Arabia; sohaib.b.habib@gmail.com; 5 Department of Pharmaceutical Care, School of Pharmaceutical Sciences, University of Phayao, Mueang Phayao 56000, Thailand; kkhanita.du@up.ac.th; 6 Colorado School of Public Health, Colorado State University, Fort Collins, CO 80523, USA; brad.reisfeld@colostate.edu

**Keywords:** machine learning, artificial intelligence, pharmacokinetics, valproic acid

## Abstract

**Background/Objectives**: Population pharmacokinetics (PopPK) has been used to aid valproic acid (VPA) dose individualization. However, this approach faces limitations owing to the complexity and high dimensionality of datasets. Machine learning (ML) can handle these challenges. However, the comparative evaluation of these ML algorithms and their practical application in optimizing VPA therapy have not yet been established. This review aims to summarize the current evidence, identify research gaps, and outline ML applications for VPA in clinical practice. **Methods**: PubMed, ScienceDirect, Scopus, the Association for Computing Machinery (ACM) Digital Library, and IEEE Xplore were searched from inception to October 2025. Eligible studies included original research articles using ML models to predict VPA pharmacokinetics or clinical outcomes (e.g., seizure control). Data on study design, population characteristics, features, predicted targets, ML algorithms, validation method, and model performance metrics were extracted. **Results**: Eleven studies were included. Most studies were retrospective, single-center designs. Ensemble tree-based models such as Random Forest, CatBoost, Gradient Boosted Regression Trees, and other ensemble methods, were consistently among the top-performing algorithms. Final models retained 3 to 19 input features, with daily dose, albumin, and body weight being the most common predictors. Only five studies performed external validation, limiting the generalizability of the models. **Conclusions**: Current VPA ML models demonstrated promising predictive performance. Nonetheless, most models are retrospective and single-center, with only limited external validation. Future VPA ML studies should use multicenter datasets and apply external evaluation to enhance model generalizability for clinical use.

## 1. Introduction

Valproic acid (VPA) or sodium valproate, a conventional antiseizure medication (ASM), is still widely prescribed around the world, especially in developing and underdeveloped countries, due to its low cost. VPA is a broad-spectrum ASM used for the treatment of absence seizures, myoclonic seizures, and generalized tonic–clonic seizures [1]. It exerts anticonvulsant properties by inhibiting voltage-gated sodium channels and T-type calcium channels [2]. In addition, VPA inhibits gamma-aminobutyric acid (GABA) transaminase, resulting in increased GABAergic neurotransmission, which in turn increases inhibitory activity [2,3,4]. Moreover, VPA has been reported to inhibit histone deacetylase, which is an enzyme associated with manic-type behavior [5]. Thus, it also plays an important role as a mood-stabilizing agent in the management of bipolar disorder [5]. The pharmacological activity of VPA also extends to other neurological conditions such as migraine and neuropathic pain [6,7].

VPA has high oral bioavailability, with values approaching 1.0 for oral solutions and capsules, whereas a bioavailability of 0.8–0.9 has been reported for sustained-release tablets [8], and it is extensively bound to albumin (90–95%) [9]. When VPA concentrations exceed 50 mg/L, binding becomes saturated, leading to a disproportionate increase in VPA concentrations [8]. Several factors, such as age, concomitant medications, and renal and hepatic diseases, may affect VPA protein binding, leading to high variability in the volume of distribution [8,10]. VPA is extensively metabolized by the liver via at least three major pathways, including cytochrome P450 (CYP450)-mediated metabolism, glucuronide conjugation, and β-oxidation in mitochondria [11,12]. The CYP450-mediated metabolism is considered a minor route (approximately 10%) [12]. CYP2C9, CYP2B6, and CYP2A6 are the key CYP450s responsible for the metabolism of VPA to 4-ene-VPA [13], while glucanosyltransferase (UGT) isoforms involved in VPA metabolism include UGT1A3, 1A4, 1A6, 1A8, and 1A9 [14]. VPA also inhibits CYP2C9; thus, it may interact with drugs that are substrates of this CYP isoform [15]. VPA is a narrow-therapeutic-index drug and exhibits high pharmacokinetic variability [16]. Thus, therapeutic drug monitoring (TDM) is crucial to achieving optimal therapeutic outcomes. The recommended VPA therapeutic range is 50–100 mg/L [8]. VPA levels above the range may be associated with gastrointestinal side effects such as nausea, vomiting, and diarrhea. Thrombocytopenia may occur in patients with VPA concentrations above 100 mg/L for females and 130 mg/L for males. When levels exceed 175 mg/L, toxicities such as central nervous system (CNS) depression, metabolic acidosis, hypernatremia, hypoglycemia, hepatotoxicity, pancreatitis, or multiorgan failure may be observed [2]. Over the past few decades, population pharmacokinetics (PopPK) combined with Bayesian estimation has become well-accepted in clinical research and specialized centers for individualizing narrow-therapeutic-index drugs. By accounting for both inter-individual and residual variabilities, this approach helps provide precise pharmacokinetic estimates for dose optimization, which is highly beneficial before steady-state conditions are achieved [17,18]. However, routine bedside implementation remains limited. In daily clinical practice, applying these models is often difficult due to the requirement for specialized software and clinicians who have a strong background in this field [19]. Moreover, PopPK models may not handle complex, high-dimensional datasets.

Recently, machine learning (ML) has emerged as a promising tool for model-informed precision dosing (MIPD) due to its ability to handle sophisticated, high-dimensional datasets [20]. Several ML models have been developed to either predict VPA pharmacokinetics or predict clinical outcomes using VPA pharmacokinetic information [21,22,23,24]. These models incorporate diverse clinical and biological features, utilize various ML algorithms and modeling methodologies, and support the optimization of VPA therapy. Maintaining VPA concentrations within the therapeutic range requires careful monitoring, as the transition from subtherapeutic to toxic exposure can occur rapidly. Concentrations below 50 mg/L may result in inadequate seizure control, while those exceeding 100 mg/L are associated with thrombocytopenia, and levels above 175 mg/L carry the risk of serious toxicities, including CNS depression and metabolic acidosis. Predicting whether VPA concentrations will fall within or outside these specific thresholds, particularly in high-risk populations, is a task for which ML models may offer advantages over conventional approaches, as they can simultaneously incorporate multiple covariates without requiring explicit pharmacokinetic model assumptions.

We selected a scoping review approach rather than a systematic review because the literature on ML applications for VPA is methodologically diverse and still developing. A scoping review is suitable for mapping the breadth of existing evidence before a more focused systematic review can be conducted. We aim to summarize and synthesize data relevant to the applications of ML models in optimizing VPA therapy. This review highlights current evidence, identifies gaps in the research, and discusses the potential application of ML models for VPA in clinical practice. To our knowledge, this is the first review to systematically map both pharmacokinetic prediction and pharmacokinetic-informed clinical outcome ML models for VPA within a single framework and to provide a quantitative, cross-study summary of their reported performance metrics, thereby offering a practical reference point for researchers designing future VPA ML studies and for clinicians evaluating the current readiness of these tools for clinical implementation.

## 2. Materials and Methods

Our review is conducted in accordance with the Preferred Reporting Items for Systematic Reviews and Meta-Analysis extension for Scoping Reviews (PRISMA-ScR) (Table S1) [25]. This scoping review was not registered in PROSPERO, as registration is not mandated for this type of review.

### 2.1. Database Searching and Selection of Relevant Studies

We systematically searched five databases, namely PubMed, ScienceDirect, Scopus, the Association for Computing Machinery (ACM) Digital Library, and IEEE Xplore, from their inception dates. Searches were last conducted on 31 October 2025, and database coverage extended to that date. We also searched reference lists for additional relevant studies. Since the primary aim of this review is to gather all available information on the application of ML to optimize VPA therapy, we defined a variety of search terms across two domains: “ML” and “valproic acid”. A comprehensive search strategy for each database is described in Table S2. We included all studies that used ML to aid VPA treatment. Studies that did not involve human participants, were not written in English, did not incorporate VPA pharmacokinetic information, or did not predict VPA pharmacokinetics were excluded. In addition, review papers and protocol-only articles were excluded. Studies that used simulated patient data generated from previously published PopPK models were eligible for inclusion, provided the simulation was designed to represent a real patient population and the resulting ML model was developed to predict VPA pharmacokinetics. We considered these studies eligible because they address the same methodological question as those using real-world data, specifically how ML can be applied to VPA pharmacokinetic prediction. However, we recognize that a model trained on simulated data may not fully capture the unpredictable variability found in actual clinical practice. Its performance primarily reflects the ability to recover patterns encoded within the baseline PopPK model, a limitation that is addressed further in the Discussion.

### 2.2. Data Extraction and Evidence Synthesis

Title and abstract screening was independently performed by J.M. and W.C. Assessment of the full-text articles based on the inclusion and exclusion criteria was also performed by the same reviewers. Any disagreements were discussed among all authors and resolved through consensus. Before synthesizing evidence across studies, we classified each included study according to its modeling objective, specifically whether the model’s target variable was a pharmacokinetic quantity (VPA concentration or dose) or a clinical outcome for which VPA concentration served as an input feature rather than the prediction target. This classification was applied prior to cross-study comparisons of features, algorithms, and performance metrics, to avoid pooling studies that address fundamentally different prediction problems.

A predesigned data extraction form developed by J.M. was used. Both J.M. and W.C. independently performed data extraction to obtain the following information: publication year, objectives, study characteristics (study design, study setting, country/region), population characteristics (sample size, population type, disease/condition of interest, demographic, clinical characteristics), ML algorithms used in each study, screened and retained features in the model, target variables, software tools, validation methods, metrics used to assess the performance of the models, and strategies used to translate the results into clinical relevance. We further explored the clinical implications of each ML model, the limitations of individual studies, and potential directions for future research in this field. Critical appraisal of the methodological quality of individual sources of evidence was not performed. This reflects the aim of this review, which was to map the breadth of ML applications for VPA therapy and characterize the features, algorithms, and reporting practices used across studies, rather than to weigh or pool evidence according to study quality. Critical appraisal is not a required component of scoping reviews [25]. All information was summarized using descriptive statistics.

We classified ML algorithms into five groups: (1) tree-based methods, (2) neural networks and deep learning, (3) kernel-based methods, (4) linear and generalized linear models, and (5) others. This classification is based on their fundamental statistical principles, algorithmic architectures, and clinical use cases to systematically evaluate their utility in pharmacometrics. The first group, tree-based methods (e.g., Random Forest (RF), Extreme Gradient Boosting (XGBoost), Categorical Boosting (CatBoost), Adaptive Boosting (AdaBoost), Light Gradient Boosting Machine (LightGBM)), operates on the principle of recursive partitioning of the feature space; its primary use case is handling tabular clinical data with complex, non-linear interactions and missing values without requiring rigid parametric assumptions [26]. The second category, neural networks and deep learning, relies on universal approximation theorems through interconnected node layers; it is best utilized in high-dimensional spaces with massive data density where automated feature extraction can uncover hidden physiological patterns [27]. The third group, kernel-based methods (e.g., Support Vector Regression (SVR)), utilizes the statistical principle of structural risk minimization and kernel mapping to project input variables into higher-dimensional spaces. This approach is ideal for smaller, clean datasets where an optimal linear hyperplane can be defined for robust regression [28]. Conversely, the fourth category, comprising linear and generalized linear models (e.g., Least Absolute Shrinkage and Selection Operator (LASSO)), functions under explicit additive linear assumptions; its principal use case is in scenarios demanding maximum model interpretability and simultaneous feature selection, achieved via *L*_1_ regularization that shrinks non-essential covariate coefficients to zero [29]. Finally, the fifth category, designated as others, includes instance-based frameworks like K-Nearest Neighbors (KNN), which operate on non-parametric memory-based principles; it is typically used in local pattern recognition, generating predictions purely based on geometric proximity or distance metrics relative to neighboring historical data points rather than constructing a global population model [30].

Model evaluation was categorized into internal and external validation [19]. A study was considered internal when the test data originated from the same source as the training data, typically created by applying various data-splitting techniques. In contrast, external evaluation was assigned to studies that assessed model performance using an independently collected dataset from a different population, clinical setting, or time frame. The model evaluation approaches and performance metrics used across the reviewed studies were systematically categorized into four primary groups: (1) confusion-based metrics to assess overall categorical classification performance, e.g., accuracy, sensitivity, and specificity, (2) threshold-based metrics to evaluate discrimination capacity across continuous cut-offs such as the area under the receiver operating characteristic curve (AUROC), (3) regression-based metrics to measure predictive errors and accuracy for continuous pharmacokinetic outcomes, e.g., Mean Absolute Error (MAE), Root Mean Square Error (RMSE), and R-square (R^2^), and (4) calibration-based metrics to determine the alignment between predicted probabilities or levels and actual clinical observations, e.g., ideal rate or calibration bands [19].

## 3. Results

### 3.1. Study Identification

**Figure 1:**
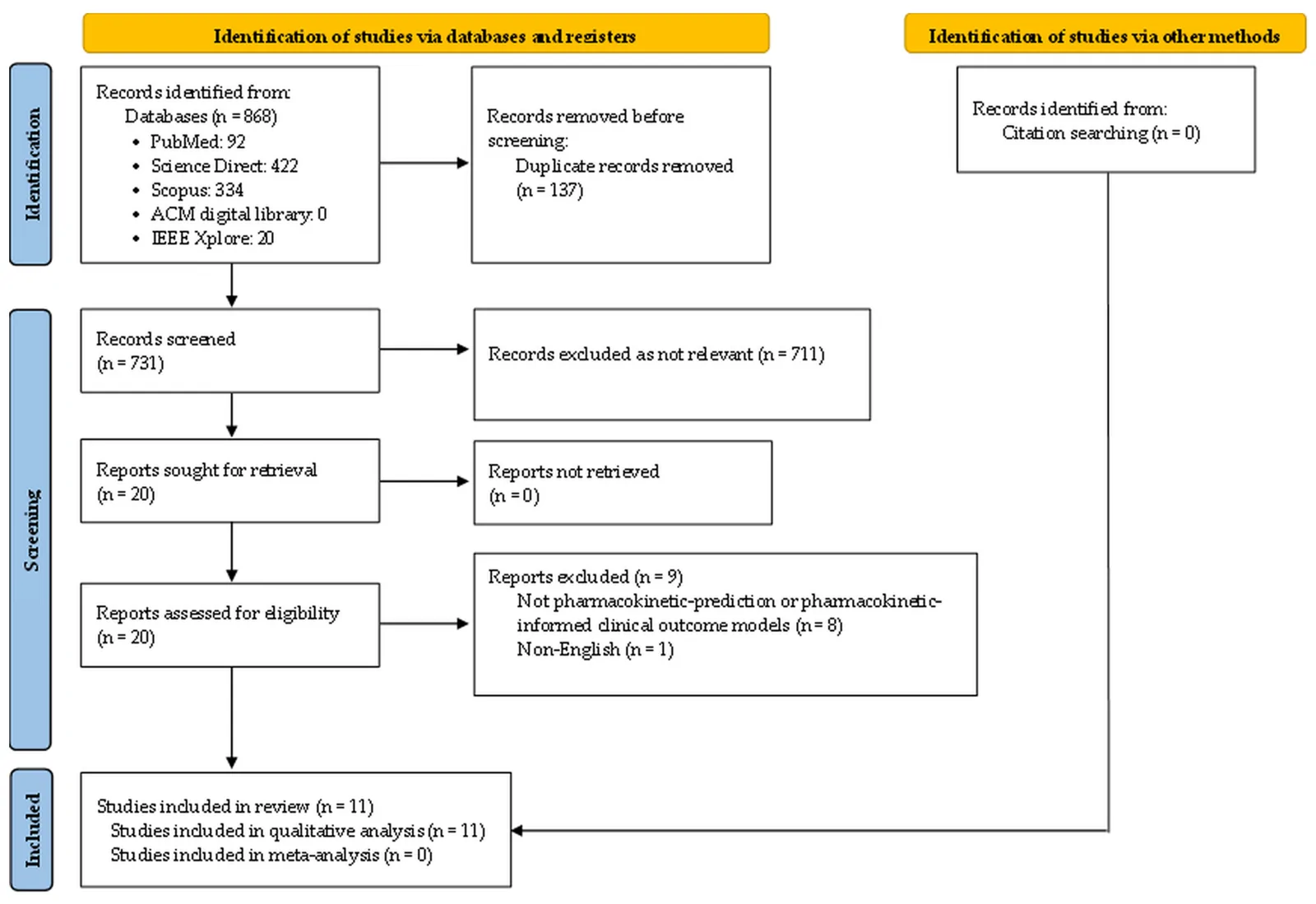
PRISMA flow diagram of study selection.

A total of 868 records were identified from the database search, and 137 duplicate records were removed, resulting in 731 records for title and abstract screening. Of these, 711 articles were excluded as irrelevant, leaving 20 for full-text screening. Subsequently, eight studies were excluded for not including pharmacokinetic information as either input features or prediction targets, and one additional study was excluded as it was not written in English. Finally, 11 studies were included in this review. A PRISMA flow diagram of the study identification is shown in Figure 1. The high exclusion rate at the title and abstract screening stage (711 of 731 records) reflects the intentionally broad search strategy, which was designed to maximize sensitivity by combining only two core domains: ‘machine learning’ and ‘valproic acid’, without restrictions by study type or outcome. The majority of excluded records were general pharmacological or clinical studies of VPA that did not incorporate ML methods, reviews, case reports, or studies involving unrelated drugs for which VPA appeared only as a concomitant medication.

### 3.2. Characteristics of the Included Studies

Almost all of the included studies developed ML models using data collected from real-world patients, although one study [31] relied exclusively on simulated datasets for model development. Most studies enrolled patients with epilepsy, except for two studies in which patients with bipolar disorder [32] and mood disorders [33] were included. Moreover, in most studies, data were collected retrospectively from medical records, while only two studies [22,34] prospectively recruited patients. Ma et al. [21] developed a model exclusively in elderly patients with epilepsy, whereas four of the included studies included only pediatric patients [22,24,34,35]. The general characteristics of the included studies are summarized in Table 1, while Table 2 presents the objectives, predictive targets, performance metrics of the best model, and key findings of each included study.

### 3.3. Features and Target Variables of the VPA ML Models

**Figure 2:**
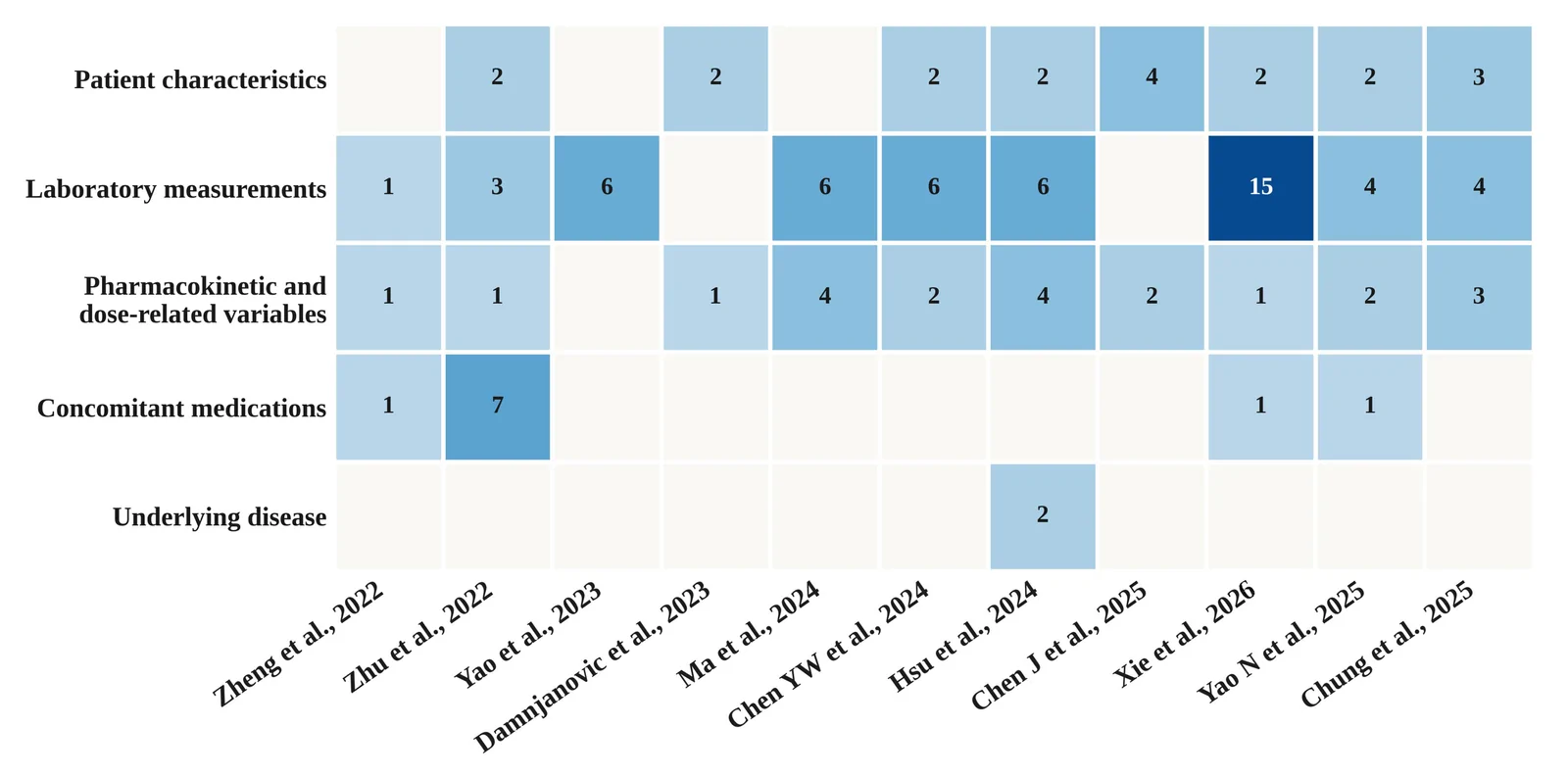
Number of retained features by category across the 11 included studies [21,22,23,24,31,32,33,34,35,36,37]. The number in each cell indicates the number of retained features in that category for the corresponding study. Darker shading indicates a higher number of retained features. Blank cells indicate that no features in that category were retained in the final model of that study.

A total of 236 features were tested among the included studies. These features were grouped into patient characteristics, laboratory measurements, concomitant medications, underlying diseases, and pharmacokinetic and dosing-related variables. Figure 2 shows the number of retained features in each category for the 11 studies; a complete list of all 236 screened and retained features is provided in Supplementary Table S3. Among patient-related features, gender (*n* = 11), body weight (*n* = 10), and age (*n* = 10) were the most commonly screened and retained in the final models. As for the laboratory measurements, albumin (*n* = 9), serum creatinine (*n* = 8), and blood urea nitrogen (*n* = 7) were the three most frequently examined features. Among these, albumin and serum creatinine were retained in the final models, while blood urea nitrogen was retained in only half of the studies that evaluated it. Platelet count, though not among the top three most frequently screened features, was ranked third among the most frequently retained features in the final models (*n* = 5). Laboratory measurements contributed the highest number of retained features overall, ranging from 1 to 15 features per study, and Xie et al. [23] retained substantially more laboratory features (*n* = 15) than any other study, which is discussed further below.

Concerning pharmacokinetic and dosing variables, total daily dose (*n* = 9), dosing interval (τ) (*n* = 4), single dose (*n* = 3), and dosing time (*n* = 3) were the most investigated features. Notably, only the total daily dose was retained in nearly all studies in the final models. Moreover, other dosing-related features—namely, time since last dose (*n* = 2), last administered dose (*n* = 2), and VPA concentrations (*n* = 2) were retained in all studies that evaluated them, despite being evaluated less frequently.

Concomitant medications were retained almost exclusively in Zhu et al. (*n* = 7) [31], while the other studies retained at most one feature in this category. Underlying diseases were retained in only one study, Hsu et al. (*n* = 2).

### 3.4. ML Algorithms, Model Evaluation, Performance Metrics, and Model Explainability

Of the five groups of ML algorithms, RF was used in all studies, followed by XGBoost, which was used in eight studies. LightGBM, CatBoost, SVR, and LASSO were each used in four studies. Among these algorithms, RF (*n* = 6) and CatBoost (*n* = 3) were most commonly identified as the top-performing final ML models. Remarkably, GBRT (*n* = 3) and ensemble models (*n* = 3), although used less frequently, also ranked among the top three best-performing algorithms. The ensemble models incorporated various combinations of algorithms, such as (1) LightGBM, CatBoost, and RF, (2) GBRT, RF, and SVR, and (3) LightGBM, GBRT, and CatBoost.

Most studies assessed the performance of their models using an internal evaluation approach with a held-out test set (6 studies) [22,32,33,35,36,37]. Five studies performed external validation. Of these, one study used external evaluation alone [36], while four studies utilized both internal and external evaluation approaches [21,23,31,34]. The most common train–test split ratio was 8:2, whereas two studies used alternative ratios of 7:3 [22] and 9:1 [33]. It should be noted that these different splitting ratios may affect the comparability of reported performance metrics across studies. A larger test set (e.g., 7:3 split) generally provides a more stable estimate of generalization errors, whereas a smaller test set (e.g., 9:1 split) may yield more variable MAE and RMSE estimates due to the limited number of observations used for evaluation. Direct comparisons of these metrics across studies should therefore be interpreted with caution.

**Table 1:** General characteristics.

Study	Sample Size	Study Design	Study Site	Participant Characteristics	Mean Age (Range) [Year]	Data Source
Zheng et al., 2022 [32]	177	Retrospective	China	Bipolar disorder patients	23 (18–29)	Real patients
Zhu et al., 2022 [31]	34,000	Simulation	China	Two PopPK models developed in Chinese epileptic patients were used for simulation	Study 1: 60 (22–88) Study 2: 26.6 (14–66)	Simulated patients
Yao et al., 2023 [35]	525	Retrospective	China	Children with epilepsy	6.42 *	Real patients
Damnjanović et al., 2023 [22]	71	Prospective	Serbia	Pediatric patients with epilepsy	10.9	Real patients
Ma et al., 2024 [21]	782	Retrospective	China	Elderly epileptic patients	67 (64–72)	Real patients
Chen et al., 2024 [34]	252	Retrospective	China	Pediatric epilepsy patients (age ≤ 14 year)	7.73	Real patients
Hsu et al., 2024 [33]	2932	Retrospective	Taiwan	Adult hospitalized patients with mood disorders receiving VPA	48.7	Real patients
Chen et al., 2025 [24]	290	Prospective	China	Pediatric patients with epilepsy	8.77 (2–18)	Real patients
Xie et al., 2026 [23]	749	Retrospective	China	Adult patients with epilepsy	45	Real patients
Yao et al., 2025 [37]	102	Retrospective	China	Chinese adult patients with epilepsy	72	Real patients
Chung et al., 2025 [36]	598	Retrospective	Korea	Patients with epilepsy receiving PHT, CBZ, PB or VPA	45.4	Real patients

PB: phenobarbital; CBZ: carbamazepine; PHT: phenytoin; PopPK: population pharmacokinetic; VPA: valproic acid. * Median.

**Table 2:** Objectives, predictive targets, performance metrics of the best model, and key findings of each included study.

Study	Objective	Predictive Target	Performance Metrics of Best Model	Remarks and Conclusion
Zheng et al., 2022 [32]	Develop an ML/deep learning model to predict VPA daily dose	VPA daily dose (0.5 g vs. 1 g)	Accuracy = 0.85, AUC = 0.91, Sensitivity = 0.85, Specificity = 0.83	CatBoost performed best. Per-class performance: Precision 55.6%/95.8%, Sensitivity 83.3%/85.2% for 0.5 g/1 g dose. Key predictors: VPA TDM value, antipsychotics, and indirect bilirubin
Zhu et al., 2022 [31]	Integrate covariates from multiple PopPK models into an ML model to predict VPA exposure	VPA steady-state concentration	Validation: MAE = 2.4 mg/L, RMSE = 3.3 mg/L, MRE = 0%, IR (±20%) = 98.85%; External (simplified model): MAE = 16.5 mg/L, RMSE = 20.1 mg/L, IR = 60.00%	XGBoost showed strong performance on simulated data integrating covariates from multiple PopPK models
Yao et al., 2023 [35]	Develop an ML model to predict VPA concentration below 50 mg/L	VPA plasma concentration < 50 mg/L	Development: AUC = 1.00, Accuracy = 1.00, Sensitivity = 1.00, Specificity = 1.00; Validation: AUC = 1.00, Accuracy = 1.00, Sensitivity = 1.00, Specificity = 1.00	The RF model predicted VPA concentrations below 50 mg/L well
Damnjanović et al., 2023 [22]	Characterize VPA, LTG, and LEV pharmacokinetics and use ML to predict seizure occurrence from drug levels and patient characteristics	Absence or Presence of seizure	Accuracy = 80.0%, Sensitivity = 75.0%, Specificity = 83.3%, Precision = 81.8%	RF predicted seizure presence/absence using VPA concentration, body weight, and gender
Ma et al., 2024 [21]	Combine ML and PopPK to predict VPA concentration	VPA steady-state concentration	Test: R^2^ = 0.740, MAE = 12.19 mg/L, RMSE = 15.29 mg/L, Abs.acc. (±20 mg/L) = 81.53%, Rel.acc. (±20%) = 75.80%; External: R^2^ = 0.631, MAE = 12.99 mg/L, Abs.acc. = 76.47%	An ensemble model (CatBoost:LightGBM:RF = 7:2:1) was combined with empirical Bayes CL/F estimates. Including PK parameters markedly improved performance compared with clinical features alone
Chen et al., 2024 [34]	Develop an ensemble ML model to monitor VPA concentration	VPA steady-state concentration	Test: R^2^ = 0.50, MAE = 9.87 mg/L, RMSE = 12.24 mg/L, Rel.acc. (±30%) = 87.8%, Abs.acc. (±15 mg/L) = 78.4%; External val 1: R^2^ = 0.41, Rel.acc. = 81.3%; External val 2: R^2^ = 0.46, Rel.acc. = 82.1%	An ensemble model (GBRT:RFR:SVR = 5:2:3) outperformed individual models.
Hsu et al., 2024 [33]	Construct predictive models (binary and continuous predictions) for serum VPA concentrations.	Insufficient treatment (concentration 1–50 mg/L) and adequate treatment (concentration 51–100 mg/L)	Continuous (RF, all features): MAE = 9.99 µg/mL, RMSE = 13.44 µg/mL, Abs.acc. (±20 µg/mL) = 88%; Binary (XGBoost, top 12 features): AUC-ROC = 0.91, Accuracy = 0.85, Sensitivity = 0.95, Specificity = 0.47	The RF model sufficiently predicted both binary and continuous outcomes
Chen et al., 2025 [24]	Compare PopPK and ML models for predicting VPA trough concentration and dosing	VPA steady-state concentration	Best ML (ResConvNN, external validation): F30 = 84%, AFE ≈ 1	PopPK alone performed poorly; MAPB improved PopPK substantially; ML and neural networks outperformed PopPK
Xie et al., 2026 [23]	Develop an optimal ML model to predict VPA concentration	VPA steady-state concentration	Test: R^2^ = 0.669, MAE = 11.11 mg/L, Abs.acc. (±20 mg/L) = 88.16%, Rel.acc. (±20%) = 64.49%; External: R^2^ = 0.621, MAE = 10.67 mg/L, Abs.acc. = 78.98%, Rel.acc. = 66.48%	An ensemble model (LightGBM, CatBoost, GBRT) yielded the best predictions
Yao et al., 2025 [37]	Develop an optimal ML model to predict VPA trough concentration in Chinese adult patients	VPA steady-state concentration	Validation: R^2^ = 0.720, MAE = 6.82 mg/L, RMSE = 9.62 mg/L, Abs.acc. (±20 mg/L) = 90.48%, Rel.acc. (±20%) = 61.90%	The Extra Tree model performed best.
Chung et al., 2025 [36]	Develop AI models to predict ASM concentrations, compare them with PopPK, and identify key covariates	VPA steady-state concentration	RF: RMSE = 13.68 µg/mL vs. PopPK (without MAPB): RMSE = 25.02 µg/mL; MAE and R^2^ not reported for VPA	ML models outperformed PopPK

Abs.acc., absolute accuracy; AFE, average fold error; AUC, area under the receiver operating characteristic curve; GBRT, gradient boosted regression trees; IR, ideal rate; LTG, lamotrigine; LEV, levetiracetam; MAE, mean absolute error; MAPB, maximum a posteriori Bayesian; ML, machine learning; MRE, mean relative error; PopPK, population pharmacokinetics; R^2^, R-squared; Rel.acc., relative accuracy; RF, random forest; RFR, random forest regression; RMSE, root mean square error; SVR, support vector regression; TDM, therapeutic drug monitoring; VPA, valproic acid.

Eight studies that predicted the continuous outcome (VPA concentration) assessed the models using regression-based metrics. Of these, MAE was the most used metric (*n* = 7), followed by RMSE (*n* = 6), R-squared (*n* = 5), and mean squared error (MSE) (*n* = 5). For studies predicting a binary outcome, confusion matrix-based and threshold-based methods were applied. The most employed metrics were sensitivity (*n* = 3), specificity (*n* = 3), and AUC-ROC (*n* = 3). Only one study utilized the GiViTI calibration band [35], and one study reported the ideal rate [31]. A summary of the model evaluation approaches and evaluation metrics is presented graphically in Figure 2.

In terms of model interpretation, all studies incorporated at least one interpretability approach, such as Shapley Additive exPlanations (SHAP), decision curve analysis, clinical-impact plots, variable-importance plots, or importance ranking, to explain or support the selection of the features in the final models. Table 3 summarizes the ML algorithms used in each study. The model evaluation methods and performance metrics used in each study are presented in Table 4.

## 4. Discussion

With rapidly growing interest in ML for MIPD, it is crucial to synthesize current evidence on key features, target variables, and ML algorithms used to develop models supporting VPA therapy. Clarifying which patient, laboratory measurement, pharmacokinetic, and dosing-related variables consistently contribute to model performance can help both researchers and clinicians understand the determinants of accurate VPA prediction and individualized dosing. Furthermore, identifying the types of targeted outcomes of the model, the algorithms that achieve the best performance, and the model interpretability approaches employed across studies provides insights into ML applications in VPA management. Such information can guide future model development and promote feature standardization and model evaluation, which ultimately supports the integration of ML-based tools into clinical practice.

We synthesized current evidence on the application of ML techniques in VPA therapy, focusing on pharmacokinetic–prediction models and pharmacokinetic-informed clinical outcome models. The majority of studies collected real-world patient data retrospectively, with only one using a simulation-based dataset. Moreover, most of the study populations consisted of patients with epilepsy, while only two studies included patients with bipolar or mood disorders [32,33]. It is well recognized that retrospective data is often limited by missing values and incomplete feature documentation, which may affect the reliability and robustness of ML models. Moreover, existing VPA ML models may not fully capture disease-specific profiles, as there was a limited number of studies involving patients with mood disorders. Future studies should extend VPA ML models to psychiatric populations and adopt prospective designs to ensure broader applicability and complete data collection. Across the 11 included studies, the overall quality of the available evidence was limited by several factors. Only five studies (45%) performed external validation, and sample sizes ranged widely from 71 to 34,000 participants, with the majority of studies enrolling fewer than 800 patients. Reporting quality also varied, as not all studies documented preprocessing procedures, hyperparameter tuning strategies, or feature selection methods in sufficient detail for full reproducibility. These observations should be considered when interpreting the findings of this review.

Most included studies developed models to predict steady-state VPA concentrations, whereas a smaller number of studies developed models to predict binary outcomes, such as seizure occurrence, treatment adequacy, or VPA concentration categories.

The preference for predicting continuous VPA concentrations rather than binary clinical outcomes is likely due to how these outcomes are measured. VPA concentration is a routine and objective marker in standard TDM practice, offering sufficient routine data without additional clinical data extraction. On the other hand, binary outcomes such as seizure control or treatment response rely on subjective clinical judgment, require longer follow-up periods, and are frequently confounded by concurrent ASM use, which increases the risk of misclassification. Furthermore, the binary-outcome studies reviewed here generally had small and imbalanced sample sizes, leading to potential model bias toward the majority class and limited generalizability. To address these issues, future work should adopt standardized definitions, collect prospective outcome data from larger multicenter cohorts, and apply class-imbalance techniques during training. Additionally, future research could integrate pharmacokinetic predictions with clinical outcomes using a two-stage approach, where predicted VPA concentrations are used as input variables for downstream clinical outcome models.

In all studies, the ML models exhibited strong predictive performance. RF, CatBoost, and XGBoost were the algorithms most frequently identified as top performers. Moreover, some studies employed ensemble models combining various algorithms, with the commonly used combinations including CatBoost, LightGBM, GBRT, and RF. SVR was included in the ensemble model in one study [34]. These ensemble models showed better performance than any of the individual algorithms used alone. The strong predictive performance of these tree-based algorithms can be explained by their ability to handle complex datasets with nonlinear relationships and feature interactions [26]. Moreover, they are resistant to overfitting and robust to noise. Altogether, these properties make them suitable for modeling the multifactorial pharmacokinetics of VPA. In contrast, SVR can model both linear and nonlinear relationships and is often suitable for sparse data, which are common in therapeutic drug monitoring studies. Nonetheless, due to its tuning complexity, its performance in larger datasets is limited [24].

Thus, ensemble models combining SVR with tree-based algorithms can help balance the strengths and weaknesses of each approach. In addition, we observed that most deep learning and neural network models did not outperform ensemble tree-based models, which could be due to the relatively limited sample sizes in the included studies, as these approaches generally require large-scale datasets to demonstrate their superiority over ensemble tree-based models.

Two studies compared the predictive performance of PopPK and ML models for VPA concentrations and found that ML models outperformed PopPK models [24,36]. In Chen et al. [24], the original PopPK model showed limited predictive performance, but performance improved substantially once maximum a posteriori Bayesian (MAPB) estimation was applied. ML and neural network models still achieved higher accuracy than PopPK, with neural networks reaching an F30 above 80%. In Chung et al. [36], the best-performing ML models showed a lower RMSE than the PopPK model for VPA (13.68 vs. 25.02 μg/mL), without MAPB estimation applied to the PopPK comparator. This suggests that the apparent advantage of ML over PopPK depends on how the PopPK comparator is implemented. When PopPK was evaluated using MAPB estimation, which reflects its intended clinical use, the performance gap with ML narrowed considerably [24]. When PopPK was applied without MAPB, ML maintained a clearer advantage [36]. However, because only two studies made this comparison, the available evidence is not sufficient to draw firm conclusions about the conditions under which ML reliably outperforms PopPK. This remains an open question for future research.

Regarding the longer-term outlook for these two approaches, we do not expect ML to completely replace PopPK modeling. Although both use statistical principles, PopPK relies on pharmacokinetic structures (such as clearance and volume of distribution) with random effects to account for patient variability. This structure allows PopPK models to simulate new dosing regimens or different patient groups, which purely data-driven ML models cannot easily do because ML depends heavily on the training data. In addition, PopPK parameters have direct pharmacokinetic meanings, making them more acceptable to regulatory agencies for dose adjustment. On the other hand, ML models, even with interpretability tools like SHAP, show statistical associations rather than pharmacokinetic parameters. Therefore, we see ML and PopPK as complementary tools. ML is useful for finding complex relationships in large datasets, while PopPK is valuable for model structure, extrapolation, and clinical dose individualization. Future studies could explore hybrid models that combine PopPK structures with ML techniques.

Several inherent technical limitations of ML in TDM should also be acknowledged. First, ML model predictions are sensitive to sampling time relative to dose administration. Inconsistent or poorly documented sampling times in clinical records may therefore introduce errors. Second, ML models cannot reliably extrapolate beyond the distribution of the training data, meaning their predictions may be inaccurate for patients whose characteristics fall outside the range represented in the training population. Third, predictions may be unstable in clinically unstable patients, such as those with acute organ dysfunction, rapid weight changes, or altered protein binding, as these conditions may not have been adequately captured in the retrospective datasets used for model development. These limitations underscore the importance of prospective validation across diverse patient populations before clinical deployment.

Several features (236) were screened across all included studies. However, only 55 unique features were retained in the final ML models of these studies. Figure 2 summarizes the number of retained features by category across studies. Gender, body weight, and age were ranked as the top three patient-related features retained in the final models across studies. This is consistent with a previous study that reported a significant influence of age and gender on VPA concentrations, in which older females required 30–50% lower VPA doses than younger females [38]. The significant impact of body weight on VPA concentrations is also consistent with several PopPK studies [16,39,40]. In terms of laboratory measurements, as expected, albumin ranked first among the most commonly retained features across studies. This was followed by serum creatinine and platelet count. VPA is highly protein-bound, and VPA concentrations, especially the free concentration, vary with albumin levels [41]. Although VPA is primarily metabolized by the liver, a small fraction of the drug can be excreted unchanged by the kidneys. Thus, elevated serum creatinine levels, which indicate impaired renal function, may reduce VPA clearance and, consequently, increase VPA concentrations [33]. Regarding platelet count, high VPA concentrations can reduce platelet levels and inhibit platelet aggregation, thereby increasing bleeding risk [42]. Nonetheless, the impact of thrombocytopenia on VPA concentrations remains unclear. This represents an important biological paradox in interpreting platelet count as a predictive feature. While thrombocytopenia may be a consequence of elevated VPA exposure, it is also retained as a predictor of VPA concentration in ML models. Whether platelet count serves as a predictor or reflects the existing VPA exposure level cannot be determined from the current cross-sectional TDM data used in most included studies. Future prospective studies with longitudinal measurements are needed to clarify the direction of this relationship.

Laboratory measurements contributed the highest number of retained features overall, and Xie et al. [23] retained substantially more laboratory features (*n* = 15) than any other study. This difference likely stems from two factors. Their dataset may simply have contained a broader set of routinely measured laboratory values. Alternatively, their feature selection process might have used less strict screening criteria, allowing variables with minor predictive value to remain in the model. Without access to the raw dataset and the exact selection process, it is difficult to determine which factor played a larger role. This highlights why future ML studies need to report their feature selection procedures in greater detail.

As one of the major limitations of ML models is their black-box nature, the impact of individual features on the target variable may not always be fully explainable. However, interpretability methods such as SHAP have been developed to identify the most influential features. Other retained features that are consistent with previous studies include total daily dose, and concomitant use of other conventional antiepileptic drugs, including carbamazepine, phenobarbital, and phenytoin. It is intuitive that patients receiving higher VPA doses would have higher VPA concentrations. While the impacts of enzyme-inducing ASMs are well documented [15,16], concomitant medications were retained almost exclusively in Zhu et al. [31], likely because that study specifically focused on enzyme-inducing ASM combinations as part of its design, rather than other studies considering comedication unimportant. Underlying diseases were retained in only one study, Hsu et al. (*n* = 2) [33], possibly because disease status is already reflected indirectly through the laboratory features included in most models, so it added little additional information once those features were accounted for.

Our review identified several limitations across studies, as follows. First, many studies were limited by small sample sizes, especially in special populations such as pediatric, elderly, or renally impaired patients. Second, only five studies performed external validation, leaving the majority of models vulnerable to overfitting and site-specific biases such as local dosing protocols or specific laboratory assays, which limits their safe generalization to different hospital settings. Third, pharmacokinetic variables differ widely between studies, in part due to inconsistent data availability across clinical settings. Direct comparisons were also challenging due to the significant variation in feature selection, outcome definitions, and data preprocessing techniques. Fourth, studies with high-dimensional features and small sample sizes might be more prone to overfitting, especially if no external evaluation is conducted. All of these drawbacks highlight the need to standardize ML development practices, particularly in feature set reporting, preprocessing, hyperparameter tuning, and model evaluation. Moreover, to enhance generalizability, future research should be conducted using large, multicenter datasets. Hybrid ML models and PopPK models with empirical Bayes estimates are also of interest, as they combine strengths from both domains. Furthermore, the use of SHAP, decision curve analysis, and other explainability tools should be encouraged to enhance model interpretability and promote clinical adoption. In terms of new features to be studied, pharmacogenomic data and other liver function markers may further improve predictive accuracy. Finally, translating ML models into clinical decision-support tools to provide real-time VPA dosing recommendations is the next important step.

This review also has limitations related to the review process itself. The search was limited to five databases and supplemented by reference list screening. Studies not published in English were excluded, as we could not verify that machine-translated versions would preserve the precision needed for extracting numerical pharmacokinetic and ML performance data. This exclusion may have introduced language bias and omitted relevant studies. Future updates of this review may address this limitation by incorporating professional translation. In addition, critical appraisal of the methodological quality of included studies was not performed. The findings therefore reflect the breadth of available evidence rather than the quality of individual studies.

## 5. Conclusions

Across the 11 included studies, VPA ML models generally showed good predictive performance for VPA concentration or dose within the populations and settings in which they were developed. However, the included studies varied widely in design, population, target variable, and evaluation rigor, and only five conducted external validation, and we did not perform a formal pooled synthesis of predictive accuracy across studies. Therefore, integrating these ML models into routine clinical practice cannot yet be recommended until they are evaluated in prospective, multicenter settings. Although some studies have reported comparable or superior predictive performance for ML relative to PopPK models, the models being compared were generally not developed using the same datasets. Additional research is required to validate these findings using the same datasets. Furthermore, hybrid model strategies, such as ensemble approaches or combinations of MAPB estimates from PopPK and ML models, may enhance their performance in individualized VPA dosing by combining the complementary strengths of different algorithms.

## Figures and Tables

**Table 3:** Machine learning algorithms used in each study.

Group		Machine Learning Methodology	Zheng P et al. [32]	Zhu X et al. [31]	Yao et al. [35]	Damnjanović et al. [22]	Ma P et al. [21]	Chen YW et al. [34]	Hsu CW et al. [33]	Chen J et al. [24]	Xie J et al. [23]	Yao N et al. [37]	Chung TK et al. [36]
Tree-based	Single tree	Decision Tree										○	○
		Decision Tree Regression		○						○			
	Bootstrap Aggregating	Random Forest	○	○	●	●	●	●	●	○	○	○	●
		Extra Tree										●	
		Extra Tree Regressor								○			
		Bagging Regression		○						○			
		Bootstrap aggregating									○	○	
	Boosting	Extreme Gradient Boosting	○	●			○		●	○	○	○	○
		Light Gradient Boosting Machine	○				●				●		○
		Gradient Boosting Decision Tree	○										
		Gradient Boosted Regression		○								○	
		Gradient Boosted Regression Trees					○	●			●		
		Gradient Boosting Machine			○								○
		Categorical Boosting	●				●				●	○	
		AdaBoost Regression		○			○			○			
		Adaptive Boosting									○	○	○
Neural Networks and Deep learning		Artificial Neural Network	○										○
		Convolution Neural Network								●			○
		Deep Neural Network								○			
		Neural Network			○								
		TabNet	○										
		Back Propagation Neural Network					○				○		
Kernel-Based Methods		Support Vector Machine	○		○				○				
		Support Vector Regression					○	●		○	○		
Linear and generalized linear models		Multiple Linear Regression		○					○			○	
		General Linear Model			○								
		Least Absolute Shrinkage and Selection Operator					○			○	○	○	
		Least Absolute Shrinkage and Selection Operator regression											○
		Elastic Net										○	
		Ridge regression									○	○	○
		Logistic Regression	○						○				
Others		Principal Component Analysis				○							
		Factor Analysis of Mixed Data				○							
		K-nearest neighbors					○				○		
		Gaussian Process Regression										○	

○ tested algorithms of each study. ● best algorithms of each study.

**Table 4:** Model evaluation approach and performance metrics used in each study.

		Zheng P et al. [32]	Zhu X et al. [31]	Yao et al. [35]	Damnjanović et al. [22]	Ma P et al. [21]	Chen YW et al. [34]	Hsu CW et al. [33]	Chen J et al. [24]	Xie J et al. [23]	Yao N et al. [37]	Chung TK et al. [36]
**Model evaluation**												
Internal evaluation (test set)		●		●	●			●			●	●
External evaluation									●			
Internal and external evaluation			●			●	●			●		
**Performance metrics**												
**Confusion-based**	Precision	●			●							
	Recall	●										
	F1-score	●										
	Accuracy	●			●							
	Sensitivity	●			●			●				
	Specificity	●			●			●				
	Negative Predictive value				●							
**Threshold-based**	Area Under the Receiver Operating Characteristics Curve (AUC-ROC)	●		●				●				
**Regression-based**	Mean Absolute Error		●			●	●	●	●	●	●	●
	Median Percentage Error								●			
	Median Absolute Prediction Error								●			
	Mean Prediction Error								●			
	Median Individual Prediction Error								●			
	Average Absolute Fold Error								●			
	Average Fold Error								●			
	Mean Relative Error (%)		●									
	Root Mean Square Error		●			●	●	●			●	●
	R-square					●	●		●	●	●	
	Mean Square Error							●	●	●		●
	Absolute accuracy					●	●	●		●	●	
	Relative accuracy					●	●		●	●	●	
**Calibration-based**	GiViTi calibration band			●								
	Ideal Rate (%)		●									

● indicates that the corresponding model evaluation approach or performance metric was used or reported in the study. AUC-ROC, area under the receiver operating characteristic curve; GiViTI, Gruppo Italiano per la Valutazione degli Interventi in Terapia Intensiva.

## Data Availability

The original contributions presented in this study are included in the article/Supplementary Materials. Further inquiries can be directed to the corresponding author.

## Supplementary Materials

The following supporting information can be downloaded at: https://www.mdpi.com/article/10.3390/pharmaceutics18081005/s1, Table S1: Preferred Reporting Items for Systematic reviews and Meta-Analyses extension for Scoping Reviews (PRISMA-ScR) Checklist, Table S2: Search terms employed in this scoping review; Table S3: Screened and retained features used in the VPA machine learning models, and their associated target variables [21,22,23,24,31,32,33,34,35,36,37].

## Abbreviations

The following abbreviations are used in this manuscript:

ASM	Antiseizure Medication
AUC-ROC	Area Under the Receiver Operating Characteristic Curve
CatBoost	Categorical Boosting
CYP450	Cytochrome P450
GABA	Gamma-aminobutyric acid
GBRT	Gradient Boosted Regression Trees
LASSO	Least Absolute Shrinkage and Selection Operator
LightGBM	Light Gradient Boosting Machine
MAE	Mean Absolute Error
MAPB	Maximum A Posteriori Bayesian
MIPD	Model-Informed Precision Dosing
ML	Machine Learning
MSE	Mean Squared Error
PopPK	Population Pharmacokinetics
PRISMA-ScR	Preferred Reporting Items for Systematic Reviews and Meta Analysis extension for Scoping Reviews
RF	Random Forest
RMSE	Root Mean Square Error
SHAP	Shapley Additive exPlanations
SVR	Support Vector Regression
TDM	Therapeutic Drug Monitoring
VPA	Valproic Acid
XGBoost	Extreme Gradient Boosting
