# Supplementary material for: Machine Learning for Valproic Acid Therapy: A Scoping Review of Pharmacokinetic-Prediction Models and Pharmacokinetic-Informed Clinical Outcome Models
Source: Pharmaceutics. 2026 Aug 14;18(8):1005. doi: 10.3390/pharmaceutics18081005 (PMC13516325; doi:10.3390/pharmaceutics18081005)
Supplement: Supplementary file 1 [file pharmaceutics-18-01005-s001.zip › pharmaceutics-4428335-supplementary.pdf]

Supplementary Materials

Table S1. Preferred Reporting Items for Systematic reviews and Meta-Analyses extension for Scoping Reviews (PRISMA-ScR) Checklist

SECTION	ITEM	PRISMA-ScR CHECKLIST ITEM	REPORTED ON PAGE #
**TITLE**			
Title	1	Identify the report as a scoping review.	Title page
**ABSTRACT**			
Structured summary	2	Provide a structured summary that includes (as applicable): background, objectives, eligibility criteria, sources of evidence, charting methods, results, and conclusions that relate to the review questions and objectives.	1
**INTRODUCTION**			
Rationale	3	Describe the rationale for the review in the context of what is already known. Explain why the review questions/objectives lend themselves to a scoping review approach.	3
Objectives	4	Provide an explicit statement of the questions and objectives being addressed with reference to their key elements (e.g., population or participants, concepts, and context) or other relevant key elements used to conceptualize the review questions and/or objectives.	3
**METHODS**			
Protocol and registration	5	Indicate whether a review protocol exists; state if and where it can be accessed (e.g., a Web address); and if available, provide registration information, including the registration number.	3
Eligibility criteria	6	Specify characteristics of the sources of evidence used as eligibility criteria (e.g., years considered, language, and publication status), and provide a rationale.	4
Information sources	7	Describe all information sources in the search (e.g., databases with dates of coverage and contact with authors to identify additional sources), as well as the date the most recent search was executed.	3
Search	8	Present the full electronic search strategy for at least 1 database, including any limits used, such that it could be repeated.	Appendix Table S2
Selection of sources of evidence†	9	State the process for selecting sources of evidence (i.e., screening and eligibility) included in the scoping review.	4

SECTION	ITEM	PRISMA-ScR CHECKLIST ITEM	REPORTED ON PAGE #
Data charting process‡	10	Describe the methods of charting data from the included sources of evidence (e.g., calibrated forms or forms that have been tested by the team before their use, and whether data charting was done independently or in duplicate) and any processes for obtaining and confirming data from investigators.	4
Data items	11	List and define all variables for which data were sought and any assumptions and simplifications made.	4
Critical appraisal of individual sources of evidence§	12	If done, provide a rationale for conducting a critical appraisal of included sources of evidence; describe the methods used and how this information was used in any data synthesis (if appropriate).	4
Synthesis of results	13	Describe the methods of handling and summarizing the data that were charted.	4
**RESULTS**			
Selection of sources of evidence	14	Give numbers of sources of evidence screened, assessed for eligibility, and included in the review, with reasons for exclusions at each stage, ideally using a flow diagram.	5, 8
Characteristics of sources of evidence	15	For each source of evidence, present characteristics for which data were charted and provide the citations.	6, 8-9
Critical appraisal within sources of evidence	16	If done, present data on critical appraisal of included sources of evidence (see item 12).	N/A
Results of individual sources of evidence	17	For each included source of evidence, present the relevant data that were charted that relate to the review questions and objectives.	9-11
Synthesis of results	18	Summarize and/or present the charting results as they relate to the review questions and objectives.	6-7, 16-17
**DISCUSSION**			
Summary of evidence	19	Summarize the main results (including an overview of concepts, themes, and types of evidence available), link to the review questions and objectives, and consider the relevance to key groups.	12
Limitations	20	Discuss the limitations of the scoping review process.	19
Conclusions	21	Provide a general interpretation of the results with respect to the review questions and objectives, as well as potential implications and/or next steps.	19

SECTION	ITEM	PRISMA-ScR CHECKLIST ITEM	REPORTED ON PAGE #
**FUNDING**			
Funding	22	Describe sources of funding for the included sources of evidence, as well as sources of funding for the scoping review. Describe the role of the funders of the scoping review.	20 (funding of the scoping review only)

Table S2. Search terms employed in this scoping review

No	PubMed	Records found
1	"machine learning"	183,452
2	"artificial intelligence"	149,847
3	#1 OR #2	298,094
4	"valproic acid"	19,253
5	"valproate"	11,886
6	"sodium valproate"	3,744
7	"divalproex"	961
8	"VPA"	7,832
9	#4 OR #5 OR #6 OR #7 OR #8	25,909
10	#3 AND #9	92
**No**	**Scopus**	**Records found**
1	"machine learning"	943,091
2	"artificial intelligence"	755,718
3	#1 OR #2	1,537,638
4	"valproic acid"	74,745
5	"valproate"	20,501
6	"sodium valproate"	4,834
7	"divalproex"	1,250
8	"VPA"	10,146
9	#4 OR #5 OR #6 OR #7 OR #8	83,405
10	#3 AND #9	334
	**ACM**	**Records found**
1	[All: "machine learning"] OR [All: "artificial intelligence"]	26,976
2	[All: "valproic acid"] OR [All: "valproate"] OR [All: "sodium valproate"] OR [All: "divalproex"] OR [All: "VPA"]	402
3	#1 AND #2	0

	IEEE Xplore	Records found
1	"machine learning"	373,352
2	"artificial intelligence"	296,451
3	#1 OR #2	611,470
4	"valproic acid"	17
5	"valproate"	7
6	"sodium valproate"	5
7	"divalproex"	5
8	“VPA”	157
9	#4 OR #5 OR #6 OR #7 OR #8	176
10	#3 AND #9	20
	**Science Direct**	
1	("valproic acid" OR "sodium valproate" OR "valproate") AND ("machine learning" OR "artificial intelligence")	422

Table S3. Screened and retained features used in the VPA machine learning models, and their associated target variables.

Category	Features	Zheng et al, 2022	Zhu et al, 2022	Yao et al, 2023	Damnjanovic et al, 2023	Ma et al, 2024	Cheng YW et al, 2024	Hsu et al, 2024	Chen J et al, 2025	Xie et al, 2025	Yao N et al, 2025	Chun et al, 2025
**Patient characteristics**	Age	○		○	●	○	●	○	●	●	○	●
	Body Mass Index	○										
	Body Surface Area								●			
	Gender	○	●	○	○	○	○	●	○	●	●	○
	Height	○				○		○	●	○	○	●
	Weight	○	●		●	○	●	●	●	○	●	●
	Diastolic blood pressure							○				
	Systolic blood pressure							○				
**Laboratory measurements**	Albumin	○	○	○		●	●	●		●	●	●
	Globulin	○					○					
	Albumin/Globulin	○				○				●		
	Total protein	○		○		●				●	○	
	Alkaline phosphatase			●		○	○	○		●	○	
	Aspartate aminotransferase	○				○	○			●	●	●
	Alanine transferase			○		○	●	○		●	○	●
	Transaminase ratio	○										
	Gamma glutamyl transferase			●		○				●	○	
	Glutamic oxaloacetic transaminase			○								
	Direct bilirubin	○										
	Indirect bilirubin	●										
	Total bilirubin	○		○		○				●		○
	Serum total bile acid			○		○				●		
	Basophil (%)			○				○				
	Basophil (absolute value)	○		○								

Category	Features	Zheng et al, 2022	Zhu et al, 2022	Yao et al, 2023	Damnjanovic et al, 2023	Ma et al, 2024	Cheng YW et al, 2024	Hsu et al, 2024	Chen J et al, 2025	Xie et al, 2025	Yao N et al, 2025	Chun et al, 2025
	Eosinophil (%)	○		○				○				
	Eosinophil (absolute value)	○		○								
	Neutrophil (%)	○				○				●		
	Neutrophil (absolute value)	○		○			○				○	
	Monocyte (%)	○		○				○				
	Monocyte (absolute value)	○		○								
	Lymphocyte (%)	○		●				○				
	Lymphocyte (absolute value)	○		○								
	White blood cell segment (%)							●				
	White blood cell (absolute value)	○		○		○	○	○		○	○	
	Red blood cell count	○		●			●	○			○	
	Hematocrit	○		○								
	Hemoglobin	○		○		●		○			○	
	Mean corpuscular volume	○										
	Mean corpuscular hemoglobin	○		○								
	Mean corpuscular hemoglobin concentration	○		○								
	Average volume of red blood cell			○								
	Red blood cell distribution width with coefficient of variation	○		○				●				

Category	Features	Zheng et al, 2022	Zhu et al, 2022	Yao et al, 2023	Damnjanovic et al, 2023	Ma et al, 2024	Cheng YW et al, 2024	Hsu et al, 2024	Chen J et al, 2025	Xie et al, 2025	Yao N et al, 2025	Chun et al, 2025
	Red blood cell distribution width with standard deviation	○		●								
	Platelet count	○		●			●	●		●	●	
	Mean Platelet Volume	○		○								
	Platelet distribution width	○		○								
	Plateletcrit/Thrombocytocrit	○		○		○						
	Platelet large cell rate	○										
	Large platelet ratio			○								
	Cystatin-C					○	○					
	Serum creatinine	○		○		○	●	●		●	○	●
	Blood Urea Nitrogen	○		○		●	●	○		●		○
	Estimated glomerular filtration rate					○				●		
	Creatinine clearance										○	
	Potassium					○						
	Sodium		○			●					○	
	Bicarbonate			○								
	Calcium							●				
	Chloride					○						
	Uric acid	○		○		●	○			●	●	
	c-reactive protein					○						
	CYP2C19 genotype *1		●									
	CYP2C19 genotype *2		●									

Category	Features	Zheng et al, 2022	Zhu et al, 2022	Yao et al, 2023	Damnjanovic et al, 2023	Ma et al, 2024	Cheng YW et al, 2024	Hsu et al, 2024	Chen J et al, 2025	Xie et al, 2025	Yao N et al, 2025	Chun et al, 2025
	CYP2C19 genotype *3		●									
	Drinking history										○	
	Smoking history										○	
	Nutritional deficiencies											○
**Pharmacokinetic and dose-related variables**	Clearance		○			●						
	Volume of distribution		○									
	Dosing interval		○			○		●			○	
	Time		○						●			○
	Time since last dose					●						●
	Daily dose		●	○		●	●	●	●	●	●	●
	Last dose							●			●	
	Single dose		○			○						●
	Dosage form			○			●					
	Dosing frequency							●				
	Total duration of treatment					●						
	Injection administration									○		
	Oral administration									○		
	Valproic concentration	●			●							
**Concomitant medications** – **Antidiabetic drugs**	Acarbose							○				
	Dipeptidyl peptidase 4 inhibitors							○				
	Glucagon-like peptide-1 agonists							○				
	Insulins							○				
	Meglitinides							○				
	Metformin							○				
	Sulfonylureas							○				

Category		Features	Zheng et al, 2022	Zhu et al, 2022	Yao et al, 2023	Damnjanovic et al, 2023	Ma et al, 2024	Cheng YW et al, 2024	Hsu et al, 2024	Chen J et al, 2025	Xie et al, 2025	Yao N et al, 2025	Chun et al, 2025
		Sodium-glucose cotransporter 2 inhibitors							○				
		Thiazolidinediones							○				
	**Cardiovascular drugs**	Angiotensin converting enzyme inhibitors							○				
		Angiotensin receptor blockers							○				
		Direct renin inhibitors							○				
		Beta blockers							○				
		Calcium channel blockers							○				
		Furosemides											○
		Diuretics							○				
		Digoxin							○				
		Amiodarone							○				
		Fibrates							○				
		Statins							○				
		Other lipid-lowering drugs							○				
		Aspirin							○				
		Clopidogrel							○				
		Ticagrelor							○				
		Other antiplatelet							○				
	**Antibiotics**	Carbapenem					○				●		
		Vancomycin											○
		Cefamezin											○
	**CNS drugs**	Alprazolam											○
		Clonazepam						○		○			○
		Lorazepam											○
		Nitrazepam								○			
		Chlordiazepam			○								
		Buspirone							○				

Category	Features	Zheng et al, 2022	Zhu et al, 2022	Yao et al, 2023	Damnjanovic et al, 2023	Ma et al, 2024	Cheng YW et al, 2024	Hsu et al, 2024	Chen J et al, 2025	Xie et al, 2025	Yao N et al, 2025	Chun et al, 2025
	Benzodiazepines					○		○				
	Non-benzodiazepines							○				
	Antianxiety drugs/tranquilizers /hypnotics	○										
	Escitalopram											○
	Bupropion							○				
	Mirtazapine							○				
	Trazodone							○				
	Agomelatine							○				
	Tricyclic antidepressants							○				
	Antidepressants	○										
	Selective serotonin reuptake inhibitor							○				
	Serotonin norepinephrine reuptake inhibitor							○				
	Lithium							○				
	Antipsychotics	●										
	Typical antipsychotics							○				
	The benzamides							○				
	The-dones							○				
	The-Pines							○				
	Aripiprazole							○				
	Acetylcholinesterase inhibitors							○				
	Memantine							○				
	Anticonvulsants and mood stabilizers	○										
	Carbamazepine		●	○				○	○			○

Category		Features	Zheng et al, 2022	Zhu et al, 2022	Yao et al, 2023	Damnjanovic et al, 2023	Ma et al, 2024	Cheng YW et al, 2024	Hsu et al, 2024	Chen J et al, 2025	Xie et al, 2025	Yao N et al, 2025	Chun et al, 2025
		Carbamazepine + Phenobarbital		●									
		Carbamazepine + Phenytoin		●									
		Carbamazepine + Phenytoin + Phenobarbital		●									
		Combination with other antiepileptic drugs										●	
		Phenobarbital		●	○		○						○
		Phenytoin		●						○			○
		Levetiracetam			○			○		○			○
		Phenytoin and Phenobarbital		●									
		Topiramate			○			○	○	○			○
		Oxcarbazepine			○			○		○			
		Clobazam								○			
		Lamotrigine						○	○	○			○
		Motriazine			○								
		Lacosamide			○					○			○
		Parampanel						○					
		Zonisamide			○								
	**Others**	Antacids							○				
		Nsaids							○				
**Underlying disease**	**Cardiovascular and vascular disease**	Aneurysm											○
		Atherosclerosis											○
		Atrial fibrillation											○
		Cardiovascular disease										○	
		Congestive heart failure							○				
		Hypertension	○				○		○			○	○
		Ischemic heart diseases											○

Category		Features	Zheng et al, 2022	Zhu et al, 2022	Yao et al, 2023	Damnjanovic et al, 2023	Ma et al, 2024	Cheng YW et al, 2024	Hsu et al, 2024	Chen J et al, 2025	Xie et al, 2025	Yao N et al, 2025	Chun et al, 2025
		Myocardial infarct							○				
		Peripheral vascular diseases							○				
	**Cerebrovascular disease**	Occlusion and stenosis of cerebral arteries											○
		Occlusion and stenosis of precerebral arteries											○
		Cerebral infarction											○
		Cerebrovascular disease	○						○			○	○
		Intracerebral hemorrhage											○
		Intracranial bleeding							○				
		Nontraumatic intracranial hemorrhage											○
		Nontraumatic subarachnoid hemorrhage											○
	**Respiratory disease**	Acute upper respiratory infections											○
		Chronic pulmonary diseases							○				
		Chronic lower respiratory diseases											○
		Influenza and pneumonia											○
	**Gastrointestinal disease**	Diseases of esophagus stomach and duodenum											○
		Intestine diseases											○

Category		Features	Zheng et al, 2022	Zhu et al, 2022	Yao et al, 2023	Damnjanovic et al, 2023	Ma et al, 2024	Cheng YW et al, 2024	Hsu et al, 2024	Chen J et al, 2025	Xie et al, 2025	Yao N et al, 2025	Chun et al, 2025
		Gastrointestinal bleeding							○				
		Intraabdominal bleeding							○				
	**Renal and urinary disease**	Chronic kidney disease	○				○					○	○
		Renal diseases							○				
		Glomerular diseases											○
		Diseases of urinary system											○
	**Endocrine and metabolic disease**	Diabetes	○				○		○			○	○
		Disorders of endocrine glands											○
		Disorders of thyroid gland											○
		Metabolic disorders											○
		Hyperlipidemia					○		○				
	**Hematological disease**	Diseases of blood											○
		Coagulation defects											○
	**Musculoskeletal disease**	Diseases of the musculoskeletal system and connective tissue											○
		Connective tissue diseases							○				
	**Neurological and psychiatric disease**	Cerebral palsy											○
		Hemiplegia							○				
		Disorders of the nervous system											○
		Inflammatory diseases of the central nervous system											○

Category		Features	Zheng et al, 2022	Zhu et al, 2022	Yao et al, 2023	Damnjanovic et al, 2023	Ma et al, 2024	Cheng YW et al, 2024	Hsu et al, 2024	Chen J et al, 2025	Xie et al, 2025	Yao N et al, 2025	Chun et al, 2025
		Neurocognitive disorders (Except vascular dementia)							○				
		Vascular dementia							○				
		Neurodevelopmental disorders							○				
		Anxiety disorders							○				
		Depressive disorders							○				
		Bipolar and related disorders							●				
		Schizophrenia spectrum and other psychotic disorders							●				
		Paraphilic disorders							○				
		Trauma- and stressor-related disorders							○				
		Feeding and eating disorders							○				
		Obsessive compulsive and related disorders							○				
		Disruptive, impulse-control, and conduct disorders							○				
		Personality disorders							○				
		Mental disorders											○
		Dissociative disorders							○				
		Elimination disorders							○				

Category		Features	Zheng et al, 2022	Zhu et al, 2022	Yao et al, 2023	Damnjanovic et al, 2023	Ma et al, 2024	Cheng YW et al, 2024	Hsu et al, 2024	Chen J et al, 2025	Xie et al, 2025	Yao N et al, 2025	Chun et al, 2025
		Episodic and paroxysmal disorders											○
		Gender dysphoria							○				
		Behavioral Therapy	○										
		Sleep-wake disorders							○				
		Somatic symptoms and related disorders							○				
		Substance-related and addictive disorders							○				
	**Oncological disease**	Any cancer							○				
		Metastatic solid tumor							○				
		Neoplasm											○
	**Hepatobiliary disease**	Diseases of liver											○
		Hepatitis	○									○	
		Hematoma or compartment syndrome							○				
		Liver diseases with sequelae							○				
		Liver diseases without sequelae							○				
		Disorders of gallbladder											○
	**Bleeding disorder**	Ocular bleeding							○				
		Other bleeding							○				
	**Infectious disease**	Acquired immune deficiency syndrome							○				

Category		Features	Zheng et al, 2022	Zhu et al, 2022	Yao et al, 2023	Damnjanovic et al, 2023	Ma et al, 2024	Cheng YW et al, 2024	Hsu et al, 2024	Chen J et al, 2025	Xie et al, 2025	Yao N et al, 2025	Chun et al, 2025
	Others	Congenital malformations of the circulatory system											○
		Congenital malformations of the nervous system											○
		Noninflammatory disorders of female genital tract											○
		Sexual dysfunctions							○				

○ investigated features

● retained features
